# The Hypoxic Microenvironment Induces Stearoyl-CoA Desaturase-1 Overexpression and Lipidomic Profile Changes in Clear Cell Renal Cell Carcinoma

**DOI:** 10.3390/cancers13122962

**Published:** 2021-06-13

**Authors:** Juan Pablo Melana, Francesco Mignolli, Tania Stoyanoff, María V. Aguirre, María A. Balboa, Jesús Balsinde, Juan Pablo Rodríguez

**Affiliations:** 1 Laboratorio de Investigaciones Bioquímicas de la Facultad de Medicina (LIBIM), Instituto de Química Básica y Aplicada del Nordeste Argentino (IQUIBA-NEA), Universidad Nacional del Nordeste, Consejo Nacional de Investigaciones Científicas y Técnicas (UNNE-CONICET), Corrientes 3400, Argentina; jpmelana@med.unne.edu.ar (J.P.M.); taniastoyanoff@gmail.com (T.S.); vikyaguirre@yahoo.com (M.V.A.); 2 Instituto de Botánica del Nordeste, Facultad de Ciencias Agrarias (UNNE-CONICET), Universidad Nacional del Nordeste, Corrientes 3400, Argentina; fmignolli80@gmail.com; 3 Instituto de Biología y Genética Molecular, Consejo Superior de Investigaciones Científicas (CSIC), 47003 Valladolid, Spain; mbalboa@ibgm.uva.es; 4 Centro de Investigación Biomédica en Red de Diabetes y Enfermedades Metabólicas Asociadas (CIBERDEM), 28029 Madrid, Spain

**Keywords:** kidney, hypoxia, tumor microenvironment, SCD-1, oleic acid

## Abstract

**Simple Summary:**

Clear cell renal cell carcinoma (ccRCC) is characterized by a high rate of cell proliferation and an extensive accumulation of lipids. Uncontrolled cell growth usually generates areas of intratumoral hypoxia that define the tumor phenotype. In this work, we show that, under these microenvironmental conditions, stearoyl-CoA desaturase-1 is overexpressed. This enzyme induces changes in the cellular lipidomic profile, increasing the oleic acid levels, a metabolite that is essential for cell proliferation. This work supports the idea of considering stearoyl-CoA desaturase-1 as an exploitable therapeutic target in ccRCC.

**Abstract:**

Clear cell renal cell carcinoma (ccRCC) is the most common histological subtype of renal cell carcinoma (RCC). It is characterized by a high cell proliferation and the ability to store lipids. Previous studies have demonstrated the overexpression of enzymes associated with lipid metabolism, including stearoyl-CoA desaturase-1 (SCD-1), which increases the concentration of unsaturated fatty acids in tumor cells. In this work, we studied the expression of SCD-1 in primary ccRCC tumors, as well as in cell lines, to determine its influence on the tumor lipid composition and its role in cell proliferation. The lipidomic analyses of patient tumors showed that oleic acid (18:1*n*-9) is one of the major fatty acids, and it is particularly abundant in the neutral lipid fraction of the tumor core. Using a ccRCC cell line model and in vitro-generated chemical hypoxia, we show that SCD-1 is highly upregulated (up to 200-fold), and this causes an increase in the cellular level of 18:1*n*-9, which, in turn, accumulates in the neutral lipid fraction. The pharmacological inhibition of SCD-1 blocks 18:1*n*-9 synthesis and compromises the proliferation. The addition of exogenous 18:1*n*-9 to the cells reverses the effects of SCD-1 inhibition on cell proliferation. These data reinforce the role of SCD-1 as a possible therapeutic target.

## 1. Introduction

Renal cell carcinoma (RCC) is the most common malignancy of the urinary system. Although the incidence of RCC has remained stable, the mortality rates have decreased by only 0.9% each year from 2007 to 2016 [1,2].

Clear cell renal cell carcinoma (ccRCC) represents the most common subtype (>80%) of RCC. The most striking phenotypic feature of ccRCC is its clear cell morphology, which has been linked to a high lipid and glycogen accumulation [3]. Neutral lipids such as triacylglycerol (TAG) and cholesterol esters (CE) are stored in prominent cytoplasmic lipid droplets (LD), which are critical for cell growth and maintenance of the cell membrane [4]. Although the presence of these droplets in ccRCC is critical for sustained tumorigenesis, their contribution to lipid homeostasis and tumor cell viability is not completely understood [5].

A ubiquitous metabolic event in cancer is the constitutive activation of the pathway for fatty acid biosynthesis. Saturated fatty acids (SFAs), monounsaturated fatty acids (MUFAs) and polyunsaturated fatty acids (PUFAs) are synthetized to sustain the growing demand for phospholipids (PLs) that are used for the assembly of new membranes, energy storage and cell signaling [6,7,8]. The activation of enzymes such as ATP-citrate lyase (ACL), acetyl-CoA carboxylase (ACC) and fatty acid synthase (FAS) leading to an increased synthesis of SFAs has been extensively studied [9,10,11,12]. SFAs later become MUFAs by the action of stearoyl-CoA desaturase-1 (SCD-1) [13]. SCD-1 is a Δ9-fatty acyl-CoA desaturase that catalyzes the insertion of a double bond in the cis-Δ9 position of several saturated fatty acyl-CoAs—mainly, palmitoyl-CoA and stearoyl-CoA—to produce palmitoleoyl- and oleoyl-CoA, respectively [14]. It has been reported that these unsaturated fatty acids affect several crucial biological functions of tumor cells, such as proliferation, signaling, invasiveness and apoptosis. It was shown that oleic acid (18:1*n*-9), one of the most prevalent free fatty acids (FFAs) in human plasma, increases the proliferation of human prostate, breast and renal cancer cells [15,16]. As noted above, it was suggested that SCD-1 could be a therapeutic target in oncology, since its pharmacological inhibition induces tumor cell apoptosis [14,17,18,19,20,21]. Controversially, it is known that certain fatty acids such as 18:1*n*-9 exert anticancer effects on many tumors, inhibiting cell proliferation and favoring apoptosis [22,23].

Solid tumors such as ccRCC often show hypoxic areas as a result of uncontrolled tumor growth, without a proper development of its associated vascular network [13]. Hypoxia inducible factors (HIF-1α and HIF-2α) are commonly stabilized key players connected to cell growth and metabolic reprogramming in ccRCC. Both factors modulate tumoral hypoxic responses through altering the cell energy metabolism, including the modification of glucose consumption [24] and the expression of a lipid metabolism-associated gene [13,25,26].

We have previously shown that SCD-1 expression correlates with other cellular markers of the tumor hypoxic microenvironment, such as EPO, EPO-R, VEGF and VEGF-R in ccRCC [27]. However, up to now, tumor hypoxia in ccRCC has not been associated with the induction of SCD-1 and its consequent modification of the tumor lipidomic profile.

In this work, we demonstrate that cellular hypoxia favors the induction of SCD-1, and this influences the cellular lipid phenotype. Furthermore, we observed that SCD-1 inhibition deprives cells of essential lipid metabolites for cell proliferation. These data provide evidence to consider SCD-1 as an exploitable therapeutic target in ccRCC.

## 2. Materials and Methods

### 2.1. Patients and Sampling Procedures

Samples of ccRCC (*n* = 12) were obtained from patients treated for radical nephrectomy in the Urology Unit of the Hospital Dr. José Ramón Vidal (Corrientes, Argentina) between 2015 and 2018. The normal distal tissues and ccRCC of the same affected kidney were surgically removed. Samples were aseptically transported to the laboratory and quickly processed. They were then fixed for histopathology and immunohistochemistry procedures.

The design and methods of this research were approved by the Bioethics Committee of the Medical Research Department at Dr. José Ramón Vidal Hospital in Corrientes, Argentina. Written informed consent was obtained from each donor. The researchers received the samples in an anonymous manner.

### 2.2. Cell lines, Proliferation and Viability Assays

Caki-1 and Caki-2 cell lines (derived from ccRCC), originally from the American Type Cell Culture Collection, were generously provided by Dr. Alfredo Martínez Ramírez (Centro de Investigaciones Biomédicas de La Rioja, Logroño, Spain) and Dr. Ricardo Sánchez Prieto (Universidad de Castilla-La Mancha, Albacete, Spain), respectively. The cells were cultured in McCoy’s 5a medium modified (Thermo Fisher, Madrid, Spain) with 10% fetal bovine serum and 50 µg/mL of gentamicin (Invitrogen, Carlsbad, CA, USA) at 37 °C under humidified conditions with 5% CO_2_. Cell proliferation and viability were measured with a Neubauer chamber and also using the CellTiter 96^®^ AQueous One Solution Cell Proliferation Assay Kit (Promega, Madison, WI, USA).

For in vitro experiments, cells were subcultured every 3 to 4 days after reaching 80–90% confluence. The cells were trypsinized, centrifuged and resuspended in the medium at a suitable density. Experiments utilizing exogenous 18:1*n*-9 were performed under serum-restrictive conditions (1%) [28].

### 2.3. Hypoxic Microenvironment

To achieve a hypoxic microenvironment similar to the tumor and the effects of HIF stabilization, Caki-2 cells were exposed to different nontoxic concentrations of CoCl_2_ in McCoy’s 5a medium modified with 1% fetal bovine serum [29,30,31,32,33,34,35]. It has been previously shown that CoCl_2_ inhibits the hydroxylation of HIF-1α, thus stabilizing HIF-1α and achieving the desired hypoxic effect [36,37,38,39].

### 2.4. Real-Time Quantitative PCR (RT-qPCR)

*SCD-1*, *HIF-1A* and *HIF-2A* mRNA were determined by RT-qPCR. Total RNA was extracted using the TRIzol reagent method (Invitrogen) according to the manufacturer’s protocols. The obtained total RNA was purified using Ambion^®^ TURBO DNA-free™. First-strand cDNA was obtained by using the Moloney murine leukemia virus reverse transcriptase (Promega) from 1 μg of RNA. qPCRs were then performed using specific primers for *SCD-1* as follows: 5′-TTCCTACCTGCAAGTTCTACACC-3′ (forward) and 5′-CCGAGCTTTGTAAGAGCGGT-3′ (reverse) with a product of 116 bp. *HIF-1A*: 5′-TGCTGGGGCAATCAATGGAT-3′ (forward) and 5′-CTACCACGTACTGCTGGCAA-3′ (reverse) with a product of 590 bp. *HIF-2A*: 5′-TATAGTGACCCCGTCCACGT-3′ (forward) and 5´-AGGGCAACACACACAGGAAA-3′ (reverse) with a product of 572 bp. *B-ACTIN*: 5′-CATGTACGTTGCTATCCAGGC-3′ (forward) and 5′-CTCCTTAATGTCACGCACGAT-3′ (reverse) with a product of 250 bp was used as the housekeeping gene.

All primers were tested for specificity using the primer BLAST program available at the National Center for Biotechnology Information website (www.ncbi.nlm.nih.gov; accessed on 1 February 2020). Cycling conditions were: 1 cycle at 95 °C for 12 min, 40 cycles at 95 °C for 15 s, 60 °C for 20 s, 72 °C for 20 s and a final extension at 72 °C for 10 min.

### 2.5. SCD-1 Inhibition Assays

CAY 10566, a potent selective SCD-1 inhibitor, was purchased from Cayman Chemical (Ann Arbor, MI, USA), dissolved in DMSO and used (3 µM) according to the manufacturer’s recommendations (noncytotoxic concentrations). Caki-2 cells were cultured in McCoy’s 5a medium modified with 1% fetal bovine serum.

### 2.6. Analyses of Fatty Acids by Gas Chromatography Coupled to Mass Spectrometry (GC/MS)

Cellular lipids were extracted using the method of Bligh and Dyer [40]. After the addition of the appropriate standards, lipids were separated by thin-layer chromatography (TLC) using as the stationary phase silica gel 60 and a mobile phase consisting of *n*-hexane/ethyl ether/acetic acid (70:30:1 *v*/*v*/*v*) [41]. Glycerolipids and glycerophospholipids were transmethylated with 500 μL of 0.5-M KOH in methanol for 30 min at 37 °C, and 500 μL of 0.5-M HCl was added to neutralize. For the transmethylation of cholesterol esters, the samples were resuspended in 400-μL methyl propionate and 600 μL of 0.84-M KOH in methanol for 1 h at 37 °C. Afterward, 50 mL and 1 mL of acetic acid and water, respectively, were added to neutralize. Analysis of the fatty acid methyl esters was carried out using an Agilent 7890A gas chromatograph coupled to an Agilent 5975C mass-selective detector operated in the electron impact mode (EI, 70 eV) equipped with an Agilent 7693 autosampler and an Agilent DB23 column (60-m length × 250-µm internal diameter × 0.15-µm film thickness) under the conditions described previously [42,43,44,45]. Data analysis was carried out with Agilent G1701EA MSD Productivity Chemstation software, revision E.02.00.

### 2.7. Confocal Microscopy

Caki-2 cells attached to coverslips were incubated for 24 h in McCoy 5a medium modified with different concentrations of CoCl_2_ and a positive control with 30-µM oleic acid. Cells were then washed with phosphate-buffered saline and incubated with BODIPY 493/503 staining solution (2 µg/mL) for 15 min at 37 °C. Cells were subsequently washed, fixed with 4% paraformaldehyde and washed again. The coverslips were mounted on slides with a DAPI reagent (1 µg/mL). Untreated cells were used as negative controls. Fluorescence was monitored by microscopy using a Bio-Rad confocal system Radiance 2100 laser scanner (Bio-Rad, Richmond, VA, USA). The images were analyzed with ImageJ software.

### 2.8. Apoptosis Detection by Flow Cytometry

The effect of SCD-1 inhibition on apoptosis was evaluated by flow cytometry. Based on the preliminary time–course data, the exposure time was set to 18 h, and apoptosis was analyzed by labeling with the annexin V-fluorescein isothiocyanate (FITC) apoptosis detection kit (BD Bioscience, San Jose, CA, USA), which recognizes phosphatidylserine exposure on the outer leaflet of the plasma membrane. After washing the cells, cell fluorescence was quantified by flow cytometry in FL1 (Gallios; Beckman Coulter, Barcelona, Spain). Data were analyzed with FlowJo software version 8.7. The propidium iodide (PI; Sigma-Aldrich, Madrid, Spain) uptake was analyzed by incubating cells with 50-µg/mL PI in PBS in the dark for 5 min. Fluorescence was quantified by flow cytometry in FL3. Data were analyzed with FlowJo version 8.7.

### 2.9. Statistics

Statistics were performed using GraphPad Prism 8.0 via an unpaired *t*-test or one-way analysis of variance (ANOVA), followed by Bonferroni’s or Tukey’s comparison tests. Differences were considered to be significant at *p* < 0.05.

## 3. Results

### 3.1. The Lipidomic Profile of ccRCC Is Dependent on the Tumor Area Analyzed

**Figure 1:**
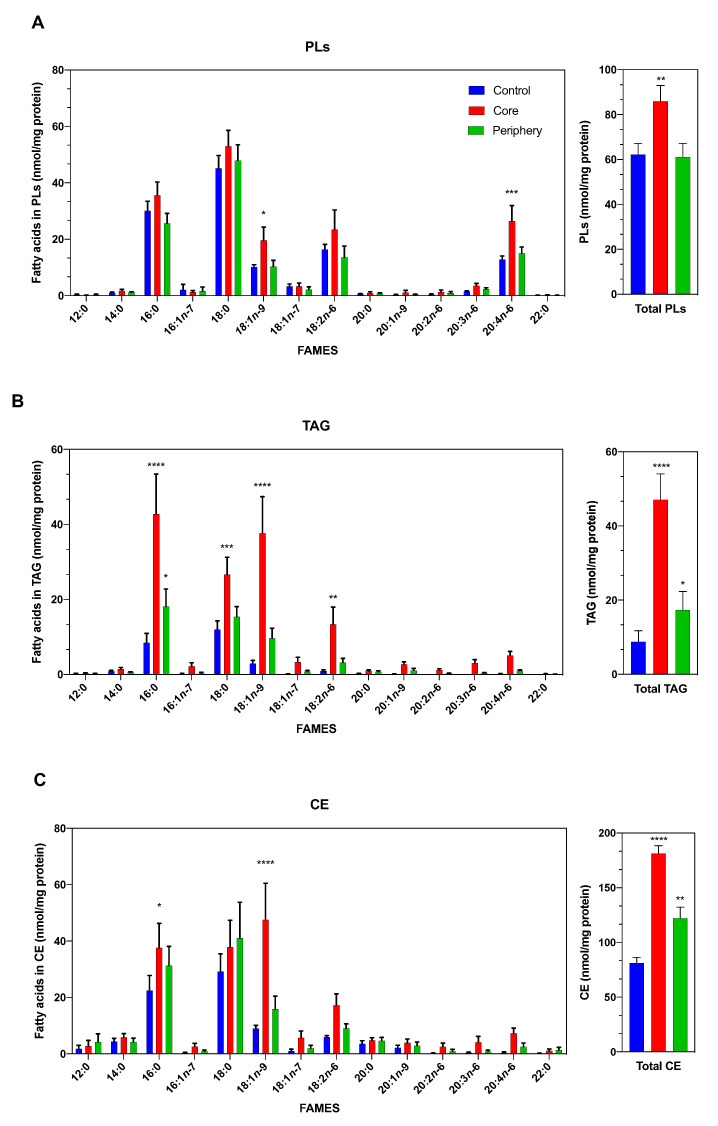
Lipidomic profile of ccRCC. (**A**) The profile of major phospholipid fatty acids in healthy distal normal tissue (blue bars) or tumors (core and periphery: red and green, respectively) were determined by GC/MS after converting the fatty acid glyceryl esters into fatty acid methyl esters. (**B**,**C**) Profile of fatty acids present in neutral lipids (TAG and CE). Data are expressed as the means ± SEM (*n* = 12). * *p* < 0.05, ** *p* < 0.01, *** *p* < 0.001 and **** *p* < 0.0001, significantly different from the control.

ccRCC tumors frequently show visible macroscopic differences with defined boundaries between the center and external areas. Therefore, we first performed a lipidomic analysis of fatty acids by GC/MS of two arbitrarily separated tumor sections: the core and periphery. Figure 1 shows that the fatty acid profile of cellular PLs did not show marked differences when the control was compared with the different tumor sections: core or periphery. Both had similar amounts and types of fatty acids, with the exception of oleic acid (18:1*n*-9) and arachidonic acid (20:4*n*-6), which were increased in the core. In contrast, the distribution of fatty acids in the TAG and CE fractions showed a higher amount of lipids in the core (Figure 1B,C). Palmitic acid (16:0), stearic acid (18:0) and, particularly, 18:1*n*-9 were greatly increased in the core compared to normal tissue or periphery. 

**Figure 2:**
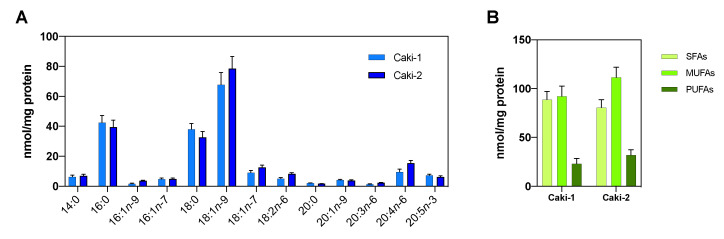
Lipidomic fatty acids profile of the Caki-1 and Caki-2 cell lines. (**A**) Total cellular fatty acid content. (**B**) Distribution of SFAs, MUFAs and PUFAs. Data are expressed as the means ± SEM and are representative of three independent experiments.

Since interindividual genotypic variations create great variability in primary cell culture models derived from tumors [46], in the following series of experiments, we used the Caki-1 and Caki-2 cell lines as an in vitro model of ccRCC. To compare the lipidomic profile of the Caki-1/-2 cell lines, we first analyzed the total cellular fatty acid content. Similar to that observed in tumors, Figure 2 shows that the most abundant fatty acids in these cell lines were also 16:0, 18:0, 18:1*n*-9 and 20:4*n*-6. Although the two cell lines showed a similar fatty acid distribution, Caki-2 had a slightly higher amount of 18:1*n*-9. While both cell lines are ccRCC models, Caki-2 was established from a primary clear cell carcinoma of the kidney, and Caki-1 was derived from a skin metastasis. Consequently, we decided to use the Caki-2 cell line for the in vitro experiments.

### 3.2. The Hypoxic Microenvironment Promotes SCD-1 Overexpression, Lipid Droplet Formation and Changes in the Cellular Fatty Acids Profile

Differences in the lipid composition, depending on the area of the tumor analyzed, should be in line with the expression pattern of the enzymes involved in their cellular metabolic pathways. Likewise, enzyme induction is strictly linked to the tumor microenvironment. Analyzing renal tumors, we previously showed that there is a statistical association of some hypoxia markers (e.g., *HIF-1A*) with the expression of *SCD-1* [27]. Thus, to investigate whether this physiological condition is actually responsible, at least in part, for SCD-1 induction, the Caki-2 cells were treated with different CoCl_2_ concentrations for 24 h to generate chemical hypoxia in vitro [30,39].

**Figure 3:**
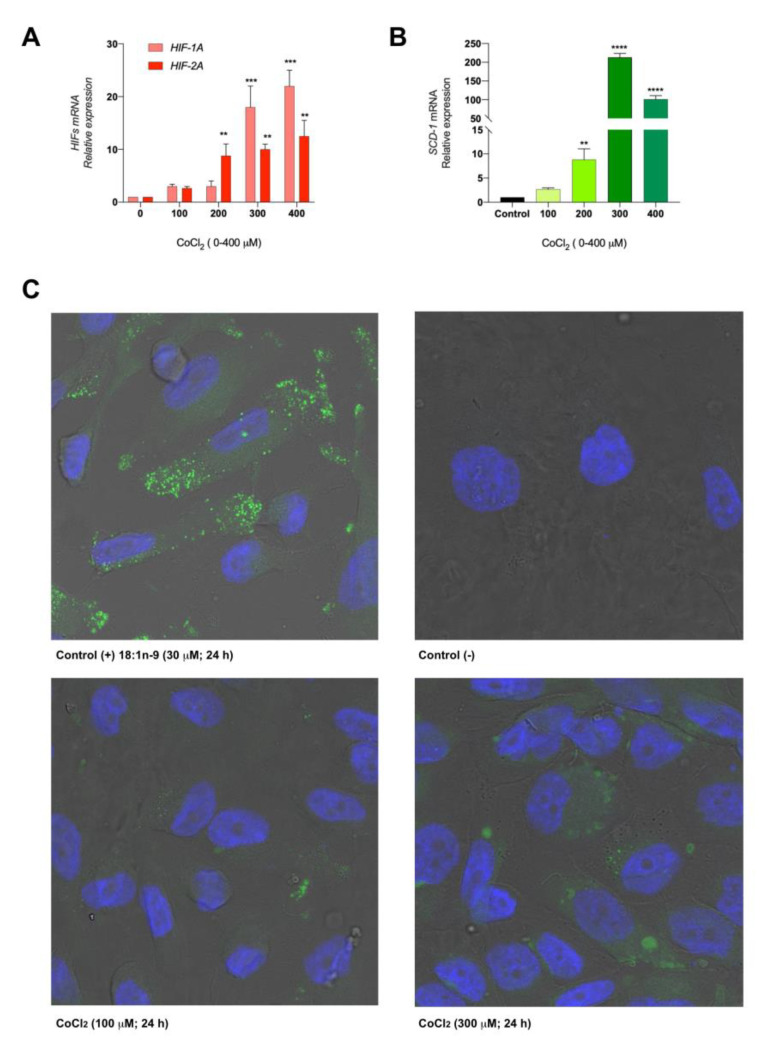
Chemical hypoxia promotes SCD-1 expression in vitro. (**A**) Caki-2 cells were exposed to different concentrations of CoCl_2_ (0–400 µM) for 24 h, and the development of the hypoxic microenvironment was tested with the expression of *HIF-1A* and *HIF-2A* by RT-qPCR. (**B**) Then, *SCD-1* overexpression was detected under the same experimental conditions by RT-qPCR. (**C**) The evaluation of LD formation was determined by confocal microscopy. Caki-2 cells were exposed to 18:1*n*-9 (30 µM) for 24 h as a positive control for LD formation. Magnification 400×. Data are expressed as the means ± SEM and are representative of three independent experiments. ** *p* < 0.01, *** *p* < 0.001 and **** *p* < 0.0001, significantly different from the control.

We first determined the cytotoxicity after the treatment with CoCl_2_ for 24 h and cell proliferation rates (growth constants (k) and cell doubling times; Supplementary Materials Figure S1). We observed that concentrations below 300 µM did not affect the cellular viability [47], but higher concentrations, up to 400 µM, induced cell death (5–10%). To verify that this salt did indeed generate cellular hypoxia, we evaluated by RT-qPCR the expression of hypoxia markers such as *HIF-1A* and *HIF-2A* (Figure 3A). Under the same conditions, the expression of *SCD-1* mRNA significantly increased (up to 200-fold) with 300-µM CoCl_2_ (Figure 3B). We hypothesized that this increase in SCD-1 would be expected to lead to elevated amounts of intracellular 18:1*n*-9, which, in turn, would induce modifications in the lipid profile and/or cytoplasmic morphological changes. Hence we next evaluated the Caki-2 cells treated with 30-µM 18:1*n*-9 as a positive control for LD formation [48] with the cells treated with CoCl_2_ (0–300 µM) by confocal microscopy. Using BODIPY^®^ staining, we detected increased LD biogenesis that was dependent on the hypoxic conditions (Figure 3C).

**Figure 4:**
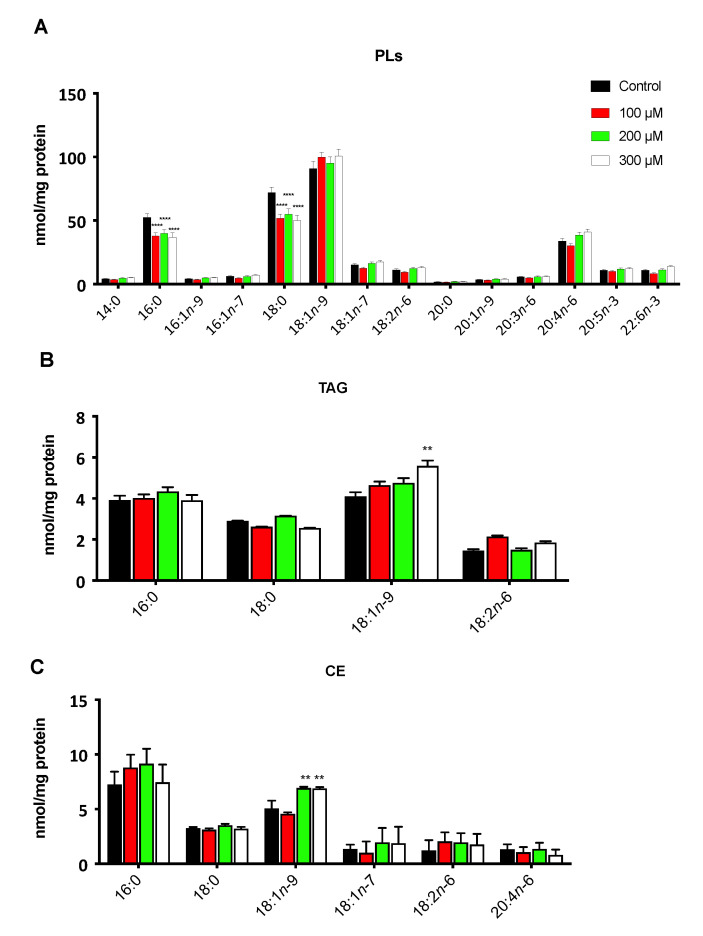
Lipidomic profile of the Caki-2 cells under hypoxic conditions. The cells were treated with the indicated concentrations of CoCl_2_, and (**A**) the fatty acid contents in the phospholipids (PL), (**B**) triacylglycerol (TAG) and (**C**) cholesterol esters (CE) were determined by GC/MS. Data are expressed as the means ± SEM and are representative of three independent experiments. ** *p* < 0.01 and **** *p* < 0.0001, significantly different from the control.

In line with these phenotypic modifications, we evaluated the hypoxia-induced lipidomic changes in Caki-2 cells using GC/MS. Notably, the saturated fatty acids (16:0 and 18:0) experienced a significant reduction (*p* < 0.001) in all treatments in the PL fraction (Figure 4A). Consistent with the induction of *SCD-1* and the appearance of cytoplasmic LD, we observed a significant increase in 18:1*n*-9 but only in the TAG and CE fractions under hypoxic conditions (*p* < 0.01) (Figure 4B,C).

### 3.3. Oleic Acid Is Essential for ccRCC Cell Proliferation

**Figure 5:**
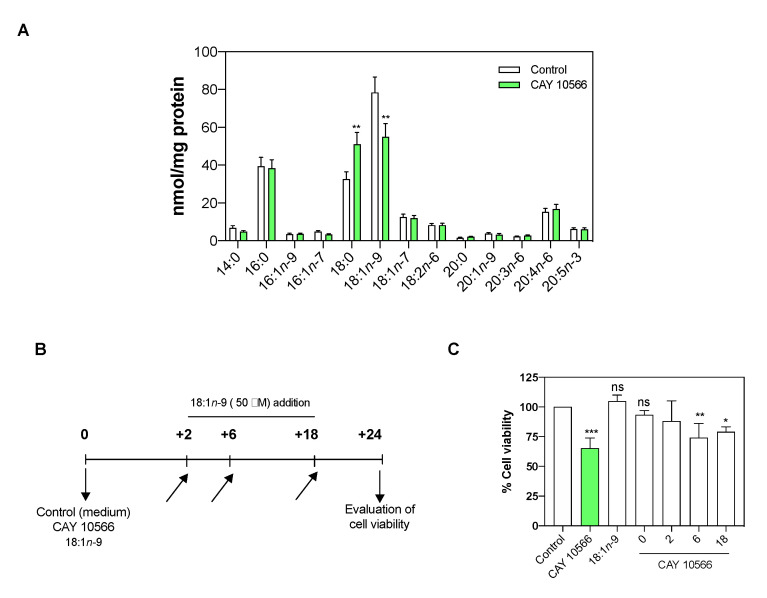
SCD-1 pharmacological inhibition. (**A**) Caki-2 cells were treated with the enzyme inhibitor CAY 10566 (3 µM) for 24 h. (**B**) Experimental design used in SCD-1 inhibition (slanted arrows indicate the addition of 18:1*n*-9 at different times). (**C**) Cell viability was measured with the CellTiter 96^®^ kit. In all cases, the cells were cultured with 1% fetal bovine serum. Data are expressed as the means ± SEM and are representative of three independent experiments. * *p* < 0.05, ** *p* < 0.01 and *** *p* < 0.001, significantly different from the control.

As additional evidence for the role of SCD-1 in the biosynthesis of MUFAs, the pharmacological inhibition of the enzyme was performed in Caki-2 cells using the selective inhibitor CAY 10566 at a nontoxic concentration (3 µM) for 24 h [49]. As shown in Figure 5A, marked lipid changes in the 18:0 and 18:1*n*-9 levels were detected. The total cellular fatty acid profile, considering all the lipid fractions simultaneously, showed a significant increase of 18:0, with a consequent decrease of 18:1*n*-9 (*p* <0.01).

In line with that demonstrated by other authors with different methodologies [50], we noted that prolonged exposure times (longer than 24 h) to the enzyme inhibitor (3 µM) induced drastic decreases in the cell viability (34.65% ± 2.97; *p*< 0.001).

If the reduced cellular levels of 18:1*n*-9 play a role in arresting the cell growth or triggering apoptosis, its addition to the cell culture would restore, at least in part, the cell proliferation. In order to check this hypothesis, 18:1*n*-9 (50 µM) was added at different times (Figure 5B,C) to Caki-2 cells with and without a treatment with CAY 10566 (3 µM). We observed that the addition of 18:1*n*-9, along with CAY 10566 or two hours after, preserved the cell viability; this effect was not observed if the fatty acid was added at later time points (Figure 5C).

**Figure 6:**
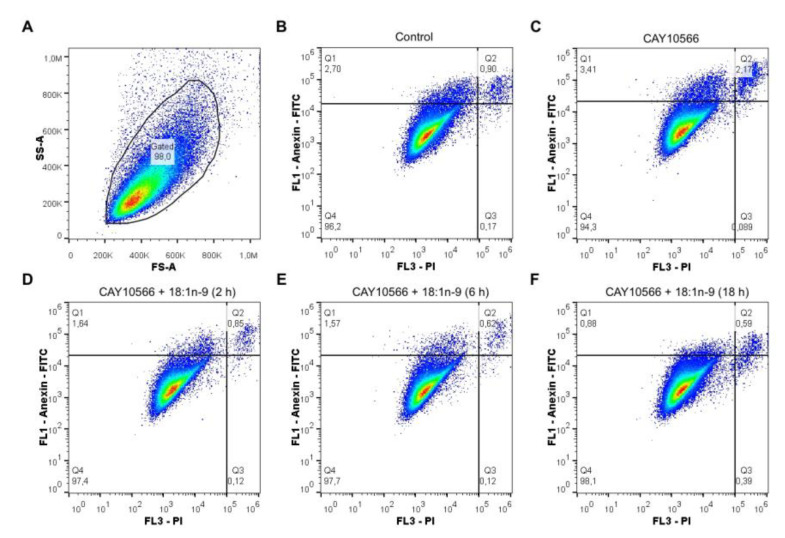
Analysis of the apoptotic markers in Caki-2 cells treated with a SCD-1 inhibitor and 18:1*n*-9. Apoptotic changes in the plasma membrane were detected by simultaneous staining with annexin V-fluorescein isothiocyanate (FITC) (FL1) and propidium iodide (PI; FL3) (**A**) Gating strategy in the control and treated cells. (**B**,**C**) Cells were untreated (control) or treated with 3-µM CAY 10566, respectively. (**D**–**F**) Afterward, 18:1*n*-9 (50 µM) was added to the cells at 2, 6 and 18 h, respectively.

In addition to evaluating the cell viability with CellTiter, we determined whether the inhibition of SCD-1 with CAY 10566 induced apoptosis in ccRCC. The Caki-2 cells were stained with annexin V-FITC and propidium iodide (50 µg/mL), as detailed in the Materials and Methods. Figure 6 shows that the cells treated with CAY 10566 manifested a small increase in apoptotic cell death, which was fully preventable if 18:1*n*-9 was present in the incubation media. Collectively, these data suggest that the decrease in cell viability that the cells experienced when SCD-1 was inhibited by CAY 10566 (Figure 5) was due to a reduced proliferation rate rather than drug-induced apoptotic cell death.

## 4. Discussion

ccRCC tumors characteristically show a bright yellow color in the center as a result of its abundant lipid content and a variegated appearance in the boundary with hemorrhage, necrosis and/or fibrosis with a frequently well-circumscribed capsule or pseudo-capsule that separates the tumor from the adjacent tissues [51]. In order to address these differences in tumor heterogeneity, we developed a separate core and periphery analysis compared to the normal distal tissues, similarly to other ccRCC-focused studies [52,53]. In all the samples individually analyzed, the highest lipid content in the core was a constant pattern, despite the interindividual differences usually determined in the oncological analysis [54,55,56]. Consistent with these results, Saito et al., developing a broader study of untargeted lipidomics, determined that these tumors have large accumulations of CE and TAG among the other lipids [53].

These imbalances are obviously associated with the particular conditions that tumor cells show to adapt their metabolism to an uncontrolled and continuous growth [57]. Several enzymes within the fatty acid biosynthesis pathway have been found to be essential for cancer cell growth or survival and are currently tracked as possible targets for therapeutic development [13,58]. Among them, SCD-1 was demonstrated to be a key regulator of the MUFA/SFA balance in several cancer cells, and its blockade triggers apoptosis [8,59,60]. In particular, SCD-1 was shown to be overexpressed in ccRCC [1] and, therefore, proposed as a possible therapeutic target for future pharmacological actions [61]. However, in none of these previous studies was enzyme inhibition associated with the lipidomic cell profile and its hypoxic context.

We previously associated the expression of cellular hypoxia markers with SCD-1 overexpression in a large number of tumor samples (*n* = 24) [27]. In this study using Caki-2 cells exposed to in vitro chemical hypoxia [47], we effectively determined that the enzyme was highly overexpressed, mimicking the microenvironmental conditions of the tumor core. In this sense, although there is evidence that SCD-1 can be modulated by post-transcriptional mechanisms involving ubiquitin proteasome-dependent and -independent pathways [62], the levels of mammalian SCD-1 appear to be principally determined by its rate of transcription [6].

Adapting to hypoxic stress is pivotal in tumor progression and determining tumor malignancy [25,63,64]. Since cellular hypoxia increases 18:1*n*-9 production, the Caki-2 cells showed increased LD biogenesis, and this was in line with the dose of CoCl_2_ used. Additionally, we used an 18:1*n*-9 overload of the same cells to simultaneously demonstrate that the 18:1*n*-9 increase is responsible for the increased LD production. The accumulation of these droplets is a sign of adaptation to stress and/or increased cell confluence [65]. The lipotoxicity that the increased synthesis of lipids brings about is neutralized, at least in part, with the production of these organelles [66,67]. LD biogenesis and degradation need to be discussed in the context of the synthesis and turnover of their major components: neutral lipids. Their synthesis is driven by the availability of precursors like TAG and CE [68,69]. Thus, we next performed a lipidomic analysis, under hypoxic conditions, using GC/MS to measure the levels of fatty acids in PLs and neutral lipids. In the PL fraction, large decreases were observed only in the SFAs (16:0 and 18:0), consequent with the increased expression of SCD-1. However, we did not observe changes at the 18:1*n*-9 level in this fraction. Conversely, the increases in 18:1*n*-9 were observed in neutral lipids, mainly in CE. Since these decreases in the SFAs are not quantitatively related to the newly formed 18:1*n*-9 by SCD-1 catalysis, most of 18:1*n*-9 must be redirected to mitochondrial β-oxidation [70] or, more likely (given the hypoxic context), released to the extracellular space. Thus, all types of cancer overexpressing this enzyme show high rates of cell proliferation, and this can only be done in cell contexts with high metabolic energy production [6,8,18,19,60,71]. On the other hand, Kamphorst et al. [72] found in other tumor lines (breast, lung and cervix) that hypoxia inhibits the catalytic activity of SCD-1 (since it uses oxygen as an electron acceptor) and shows that 18:1*n*-9 could be imported from the extracellular space.

The essential role of SCD-1 in cancer cell mitogenesis was unambiguously demonstrated by several works in which the suppression of SCD-1 by genetic and pharmacological means led to a slower rate of cell proliferation and decreased survival [61]. In this study, we observed that the viability of CAY 10566-treated Caki-2 cells was strongly correlated with the degree of inactivation of SCD-1, firmly establishing a positive relationship between the rate of MUFA synthesis and cell replication. Thus, at different times, we restored 18:1*n*-9 in the cell culture and observed that the cell viability improved, compared with CAY 10566 -treated Caki-2 cells. Taken together, the lipidomic profile and cell viability experiments allowed us to assume that changes in the 18:1*n*-9 levels are critical for these tumor cells. Simultaneously, it has been observed that the excess content of long-chain fatty acids, especially SFAs, triggers programmed cell death in a process known as lipid-mediated toxicity or lipoapoptosis [73]. Thus, these two effects (SFA increase and 18:1*n*-9 decrease, caused by SCD-1 inhibition) evidently synergize and explain the cytotoxicity observed by long-term pharmacological inhibition.

Finally, the findings described here support the concept that SCD-1 may be a potentially useful target for ccRCC treatments [61,74]. The specific design of small-molecule inhibitors of SCD-1 activity could be of great potential for possible therapeutic agents [75]. Likewise, the association of SCD-1 inhibitors with therapeutic agents that target signaling pathways and their receptors (i.e., tyrosine kinase-mediated cascades, such as pazopanib, sunitinib, axitinib and cabozantinib, or temsirolimus, which targets mTOR, among others) already in use in medical oncology could also be an attractive option for future implementation [2]. Despite these hypothetical considerations, establishing the value of SCD-1 inhibitors as a protective agent for the treatment of ccRCC will require more extensive experimental testing and careful preclinical validation.

## 5. Conclusions

In this work, we provided evidence supporting the hypothesis that the lipid composition of ccRCC depends on the hypoxic microenvironment prevailing in certain areas, such as the center of the tumor. Our results added to this concept by demonstrating that there is a tumor microheterogeneity in terms of the fatty acid distribution in different lipid species such as PLs, TAG and CE. In line with the above, we showed that SCD-1 is particularly influenced by hypoxia, since it is overexpressed under these conditions and catalyzes the conversion of 18:0 into 18:1*n*-9, favoring tumor cell proliferation. In addition, we provided evidence to reinforce the idea that SCD-1 is a meaningful pharmacological target pondering the global hypoxic context of the tumor microenvironment.

## Data Availability

The datasets used during the current study are available from the corresponding author upon reasonable request.

## Supplementary Materials

The following are available online at https://www.mdpi.com/article/10.3390/cancers13122962/s1: Figure S1: Cell proliferation rates (growth constants (k) and cell-doubling times) in all the conditions tested. Data are expressed as the means ± SEM and are representative of three independent experiments.
