# Supplementary material for: The Hypoxic Microenvironment Induces Stearoyl-CoA Desaturase-1 Overexpression and Lipidomic Profile Changes in Clear Cell Renal Cell Carcinoma
Source: Cancers (Basel). 2021 Jun 13;13(12):2962. doi: 10.3390/cancers13122962 (PMC8231571; doi:10.3390/cancers13122962)
Supplement: Supplementary file 1 [file cancers-13-02962-s001.zip › cancers-1200721-supplementary.pdf]

cancers

MDPI

Supplementary Materials: The Hypoxic Microenvironment Induces Stearoyl-CoA Desaturase-1 Overexpression and Lipidomic Profile Changes in Clear Cell Renal Cell Carcinoma

Juan Pablo Melana, Francesco Mignolli, Tania Stoyanoff, María V. Aguirre, María A. Balboa, Jesús Balsinde and Juan Pablo Rodríguez

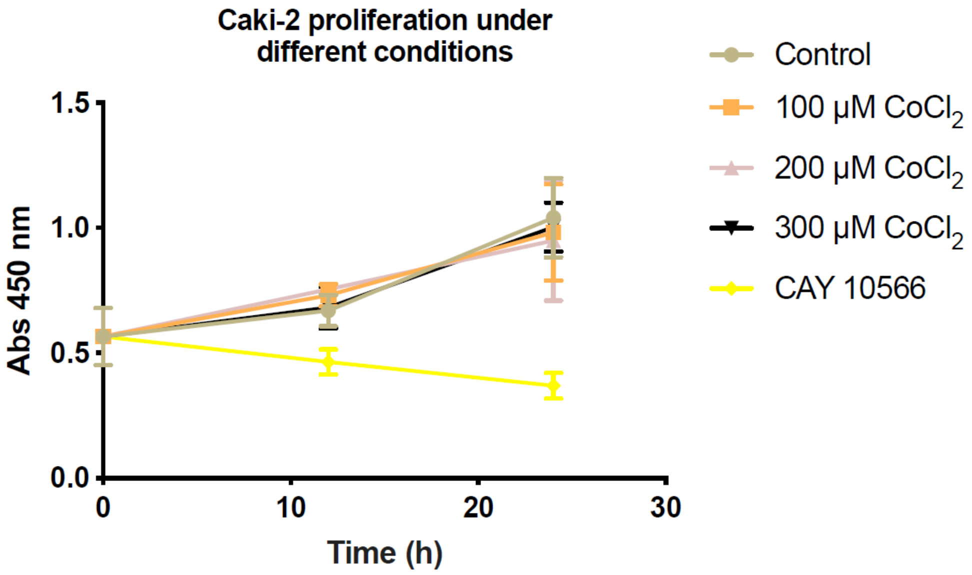

	Control	100 µM $CoCl_2$	200 µM $CoCl_2$	300 µM $CoCl_2$	CAY 10566
k	0.028	0.023	0.021	0.025	-0.017
Doubling Time (h)	24.69	29.57	32.89	26.91	-39.07

Figure S1. Cell proliferation rates (growth constants (k) and cell-doubling times) in all the conditions tested. Data are expressed as the means ± SEM and are representative of three independent experiments.
